# The Effects of Exercise on Natriuretic Peptides in Individuals without Heart Failure

**DOI:** 10.3390/sports4020032

**Published:** 2016-05-31

**Authors:** Hidetaka Hamasaki

**Affiliations:** 1 Department of Internal Medicine, National Center for Global Health and Medicine Kohnodai Hospital, 1-7-1 Kohnodai, Chiba 272-8516, Japan; hhamasaki78@gmail.com; Tel.: +81-047-372-3501; Fax: +81-047-372-1858; 2 Hamasaki Clinic, 2-21-4 Nishida, Kagoshima 890-0046, Japan

**Keywords:** exercise, natriuretic peptide, atrial natriuretic peptide, B-type natriuretic peptide, metabolic disease

## Abstract

Cardiac natriuretic peptides (NPs) play an important role in the regulation of energy expenditure in skeletal muscle and adipose tissue. A systematic review on the effects of exercise on NPs in patients with heart failure reported that aerobic and resistance training reduced NPs; however, the effects of exercise on NPs and the underlying mechanism of exercise-induced NP secretion in subjects without heart failure remain unknown. In athletes and young, healthy subjects, the NP concentration at rest is not elevated, but strenuous endurance exercise significantly increases NPs. The exercise-induced increase in NPs may be caused by transient myocardial wall stress, cardiomyocyte metabolic changes, or neuroendocrinological response, which may have cytoprotective and growth-regulating effects on the heart. On the other hand, in elderly, overweight/obese subjects, and patients with hypertension, NP concentrations also increase during exercise; however, NP secretion may be more susceptible to cardiac stress compared to young, healthy individuals. Recent studies have shown that NPs are associated with thermogenesis in fat tissue and oxidative capacity in skeletal muscles. NPs may also have a protective role for skeletal muscle in humans, although further studies are warranted to elucidate the physiological mechanism of exercise-induced NP secretion.

## 1. Introduction

**Figure 1:**
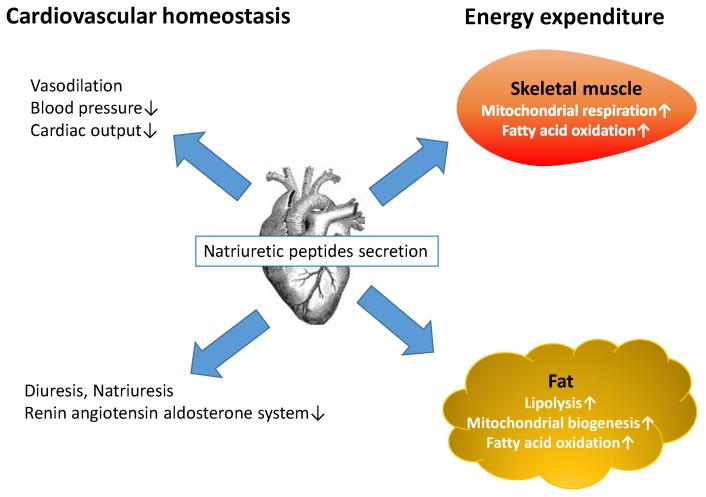
Natriuretic peptides (NPs) released by the heart have various physiological functions. NPs reduce blood pressure, cardiac burden, and renin-angiotensin-aldosterone system activity and increase renal sodium and water excretion. Moreover, NPs enhance lipolysis in human adipose tissue and increase fatty acid oxidation in both adipose tissue and skeletal muscle. NPs also enhance mitochondrial respiration in skeletal muscle and mitochondrial biogenesis in white adipose tissue, leading to increased energy expenditure in humans.

Cardiac natriuretic peptides (NPs) including atrial natriuretic peptide (ANP), B-type natriuretic peptide (BNP), and C-type natriuretic peptide (CNP) play an important role in the regulation of cardiac function [1]. NPs have vasodilator and diuretic effects, leading to decreased circulating blood volume, arterial pressure, central venous pressure, and cardiac output [2]. Plasma BNP and N-terminal proBNP (NT-proBNP) have become useful biomarkers for the diagnosis and prognosis of heart failure in clinical practice [3,4,5,6,7]. In addition, their levels are inversely associated with visceral fat [8], body mass index, waist circumference, and serum insulin levels [9,10,11,12], suggesting that obesity may be ameliorated by the lipolytic effects of BNP and NT-proBNP [13]. Previous studies have shown that ANP is also a lipolytic hormone that increases free fatty acids and enhances oxidation in skeletal muscle, liver, and adipose tissue [14,15,16]. Recently, a number of studies have suggested the CNP and its specific receptor, natriuretic peptide receptor B play an important role in regulating cardiovascular system and endothelial functions, but its physiological role has not been fully investigated [17]. For example, a randomized study suggested that the change in CNP concentrations after aerobic physical training represent an improvement of endothelial function in patients with heart failure [18]. NPs play a key role in regulating energy expenditure and fat metabolism as well as cardiovascular homeostasis (Figure 1) [19]. 

A systematic review of randomized controlled trials (RCTs) investigating the effects of exercise on BNP and NT-proBNP in patients with heart failure showed that aerobic and resistance training had a favorable effect on NPs and decreased BNP levels (mean difference −79 pg/mL; 95% confidence intervals (CI), −141 to −17 pg/mL) and NT-proBNP levels (mean difference −621 pg/mL; 95% CI, −844 to −398 pg/mL) [20]. However, this analysis was among patients with heart failure; the effects of exercise on NPs in subjects without heart failure, especially in older individuals and/or patients with metabolic diseases, have not been fully elucidated. Exercise such as long distance running [21], cycling [22,23], and hand grip exercises [24] have been reported to increase levels of NPs in healthy subjects. Recently, low-intensity daily physical activity was also suggested to be associated with increased plasma BNP levels in patients with type 2 diabetes [25,26]. A cross-sectional study showed that plasma BNP levels were inversely associated with both visceral fat area and thigh muscle mass [27], suggesting that BNP levels increase in response to muscle loss (e.g., sarcopenia) to protect against muscle damage [27]. Larsen *et al.* reported that muscle fiber roundness, an indicator of histological skeletal muscle damage because of intramuscular edema, is positively correlated with NT-proBNP levels [28]. The crosstalk between NPs, fat tissue, and skeletal muscle in humans should be studied to reveal the various physiological actions of NPs.

This review is aimed at summarizing clinical studies investigating the effects of exercise on NPs, specifically ANP and BNP, concentrations in subjects without heart disease and at providing clinicians with current knowledge on the roles of NPs in humans.

## 2. Methods

The author searched the literature on NPs and exercise using Pubmed from its inception to March 2016. The search terms were “natriuretic peptide” and “exercise.” The search returned 1402 published articles; however, 1261 articles were related to heart diseases, and they were excluded from this review. The titles and abstracts of the identified articles were reviewed to determine their relevance. If the study participants had heart diseases such as heart failure, coronary artery disease, and myocardial infarction, the studies were excluded from this review. Not only controlled trials but also before-after studies, as well as cross-sectional studies, were reviewed. A total of 18 articles were eligible. 

## 3. The Effects of Exercise on NPs in Athletes and Healthy Subjects

To the best of the author’s knowledge, no RCTs have investigated the effects of aerobic and/or resistance exercise on NPs in subjects without heart diseases. However, some non-randomized and non-controlled clinical studies have found changes in the levels of NPs due to exercise. Ohba *et al.* recruited 10 amateur male athletes (mean age = 46.2 ± 10.7 years) who participated in a 100-km ultramarathon. Their levels of plasma ANP and BNP increased two- and five-fold, respectively, immediately after running [29]. Interestingly, plasma ANP levels decreased in two athletes (23 and 36 years, respectively), but not in any older subjects. The release of ANP after exercise was greater in older subjects than in young individuals [30], and this study also suggested that the pattern of ANP secretion after prolonged strenuous exercise is related to age. Similarly, Siegel *et al.* measured changes in BNP in 82 runners without smoking habits or coronary heart disease (mean age = 47 ± 8 years) after the Boston Marathon [31]. The results showed that BNP levels did not increase 4 h after running, but significantly increased within normal limits 24 h after the race. However, other cardiac markers such as creatinine kinase-MB and cardiac troponin I were significantly increased at both 4 and 24 h after running. These results suggest that BNP secretion due to endurance exercise cannot be attributed only to exercise-related myocardial damage. Neumayr *et al.* measured NT-proBNP as a marker of cardiac injury in 29 recreational cyclists to investigate whether strenuous exercise induced cardiac dysfunction [22]. The NT-proBNP levels of study subjects without cardiovascular risk factors (mean age = 34 ± 8 years) were measured after they finished the cycling race (total distance = 230 km, altitude difference = 5500 m). The plasma levels of NT-proBNP significantly increased immediately after the race, decreased on the following day, and returned to baseline values after one week. The increase of NT-proBNP may be related to the physiologic endocrine response against the myocardial stress induced by exercise, which is not so much cardiac injury as cardiac fatigue. A study with a larger number of subjects was conducted to examine the increase of NT-proBNP and its relationship to the increases in cardiac troponin I and T after prolonged strenuous exercise [32]. A total of 105 healthy endurance athletes (mean age = 40 ± 8 years) were investigated at three endurance exercise events (a marathon, a 100-km ultramarathon, and a mountain bike marathon). The NT-proBNP levels increased in the participants after all three exercise events and exceeded the upper reference limit in 81 athletes 3 h after endurance exercise. The increases in NT-proBNP were not associated with increases in cardiac troponin I and T; however, they were related to exercise time, suggesting that BNP secretion during and after exercise is not caused by myocardial damage, but instead by cytoprotective and growth-regulating effects, as previously reported in experimental studies [33,34]. NPs have a role as anti-inflammatory and antioxidant regulators inhibiting the induction of inflammatory mediators such as tumor necrosis factor-α, interleukin-1, monocyte chemoattractant protein-1, nitric oxide, cyclooxygenase-2, and reducing oxidative stress [35,36]. Exercise may trigger such cytoprotective effects of NPs. 

Scherr *et al.* investigated the 72-h kinetics of cardiac biomarkers such as high-sensitive cardiac troponin T, heart-type fatty acid-binding protein, and NT-proBNP, inflammation markers, and renal function in marathon participants [37]. A total of 102 healthy men (mean age = 42 ± 9.5 years) were recruited, and cardiac biomarkers were measured before the race and 0, 24, and 72 h after the marathon. The NT-pro BNP levels increased significantly at 0, 24, and 72 h after the race compared to one week before the race. The NT-proBNP and heart-type fatty acid-binding protein kinetics showed similar patterns after the race. Other cardiac biomarkers increased immediately after the race but returned to normal levels within 72 h. These results suggest that the increase in cardiac markers due to endurance exercise may not be explained by myocardial damage but by transient stress or altered cardiomyocyte metabolism. To confirm the evidence of exercise-induced myocardial damage, Scharhag *et al.* conducted two endurance exercise trials in healthy athletes [38]. Twenty male athletes (mean age = 36 ± 7 years) performed both 1 h of exercise at an intensity of 100% of the individual’s anaerobic threshold and 3 h of exercise at 75% intensity (running or mountain biking). Myocardial damage was evaluated by echocardiography and magnetic resonance imaging. The levels of cardiac troponin I- and NT-proBNP significantly increased after both exercise sessions, whereas the cardiac troponin T levels did not change. No significant correlations were observed between the exercise-induced increase in NT-proBNP and cardiac troponins. Moreover, no evidence for myocardial damage was detected in echocardiography or magnetic resonance imaging. Considering the absence of relationships among NT-proBNP, cardiac troponins, and cardiac function, the exercise-induced increase in cardiac markers including NT-proBNP may represent a physiological reaction in cardiomyocytes without pathological damage. However, the cardiac effects of such physiological elevation of NT-proBNP remain unknown. Faviou *et al.* investigated whether the release of NT-proBNP after exercise had cytoprotective effects or was caused by myocardial damage during exercise [39]. A total of 99 subjects were assigned to three groups: 43 male soccer and basketball players (mean age = 24 ± 4 years); 21 age-matched patients with left ventricular hypertrophy or impaired left ventricular function; and 35 age-matched healthy controls. The athlete group performed regular training for 11–18 h per week, and the control group engaged in exercise occasionally (2–3 times a week) for 3–6 h per week. The cardiac troponin T and NT-proBNP levels at rest were measured in all three groups. After intense exercise, the changes in cardiac troponin T and NT-proBNP in the athlete group were examined. Cardiac function was evaluated by electrocardiography and echocardiography in all participants. The NT-proBNP levels in patients were significantly higher than those in the healthy controls and athletes. After intense exercise, the NT-proBNP levels were elevated compared to the levels at rest, although they were lower than those in patients at rest. In athletes, cardiac troponin T did not change after exercise. The elevation of NT-proBNP within the normal limit may not represent cardiac dysfunction; instead, it likely indicates the activation of a counter-regulatory system (e.g., the adrenergic system). NPs may be considered as an indicator of the neuroendocrine system rather than a marker of cardiac dysfunction [40]. To date, human and experimental animal studies have shown that various neuroendocrine alterations occur as a result of exercise stress and negative energy balance caused by strenuous and repetitive exercise (Table 1) [41,42]. Hypothalamic-pituitary-adrenocortical axis, antidiuretic hormone and NPs responses to exercise may contribute to integrative regulation of cardiovascular homeostasis. 

An interesting open-labeled, randomized, crossover study investigating the ANP response to exercise in water was conducted in Germany [43]. Seventeen healthy men (mean age = 31 ± 3.6 years) were studied. The subjects performed two exercise tests on a bicycle ergometer on land and in water. Exercise in water attenuated norepinephrine and epinephrine concentrations during peak exercise in comparison to exercise on land. On the other hand, the increases in ANP levels were significantly augmented when subjects exercised in water, particularly at the anaerobic threshold. Water immersion causes physiological changes such as intracellular-intravascular fluid shifts and increased cardiac output, resulting in an increase in circulating blood volume [44]. Exercise in water may stimulate ANP secretion not by the activation of sympathetic nervous system but by such physiological changes in water. When discussing the effects of exercise on NPs, we should also note the conditions under which exercise is performed (e.g., on land, in water, outside, inside, a high place, or underground). Table 2 shows a list of published articles investigating the effects of exercise on NPs in athletes and healthy subjects. These studies in athletes have focused on whether NPs reflect myocardial damage because several cases of sudden death due to cardiovascular causes have been reported in athletes. However, recent studies suggest that NPs also play a role in mediating the metabolic and endocrine systems in individuals with low levels of physical fitness [2]. This review focuses on the associations between NPs and metabolism in non-athletes, older people, and patients with metabolic disturbance.

## 4. The Effects of Exercise on NPs in the Elderly and in Patients with Metabolic Diseases Who Do Not Suffer from Heart Failure

Evidence related to the effects of exercise on NPs in non-athletes without heart failure is limited. Engelmann *et al.* investigated the responses of plasma ANP and BNP to exercise (cycle ergometer) in patients with atrial fibrillation and in healthy control subjects [45]. As expected, the plasma ANP and BNP levels at rest, during exercise, and after exercise were significantly higher in patients with atrial fibrillation than in the control subjects. The mean age of the control group was 67.9 ± 4.5 years, which is relatively high compared to the ages of athletes in clinical studies. During exercise, the ANP and BNP levels significantly increased in elderly, healthy subjects. Interestingly, no changes in ANP and BNP levels were observed between the peak exercise levels and the levels 30 min after exercise in the healthy controls. However, the decrease in plasma BNP in patients with atrial fibrillation was significantly higher than that in healthy controls, whereas the decrease in plasma ANP did not differ between the two groups. These results suggest that BNP may respond rapidly to exercise-induced left ventricular overload as an emergency hormone. 

Tanaka *et al.* measured the plasma ANP and BNP levels in 19 patients with hypertension (mean age = 44 ± 2.6 years) and 14 normotensive subjects (mean age = 40 ± 2.2 years) during exercise using a bicycle ergometer [46]. The plasma ANP and BNP levels, blood pressure, heart rate, and plasma norepinephrine levels significantly increased during exercise in both hypertensive and normotensive subjects. Norepinephrine was a significant stimulus for ANP secretion in normotensive subjects, while the changes in ANP levels were associated only with heart rate in hypertensive patients. On the other hand, systolic blood pressure and epinephrine levels were strongly associated with BNP secretion in normotensive subjects, while the changes in BNP levels were associated only with heart rate in hypertensive patients. These results suggest that exercise-induced NP secretion is more sensitive to sympathetic activity in normotensive subjects compared to in patients with hypertension. Thus, in patients with hypertension, the increase in NPs during exercise may represent cardiac stress rather than neuroendocrinological response, which is a potential mechanism of exercise-induced NP secretion in healthy athletes. 

A prospective, randomized study assessed the proANP concentrations at rest and after incremental exhaustive exercise in overweight and obese patients [47]. A total of 125 overweight and obese but otherwise healthy subjects were recruited. The 75 subjects that lost more than 5% of their weight during the six-month dietary intervention were analyzed. The subjects performed a stepwise incremental exercise test (bicycle ergometer) until exhaustion. After six months of dietary weight reduction with a low-fat or low-carbohydrate diet, the proANP levels did not change; however, exercise-induced acute increase in proANP levels were observed during exercise both before and after dietary intervention. In addition, natriuretic peptide receptor C (NPR-C) expression in abdominal fat tissue significantly decreased after the six-month hypocaloric diet intervention. These results indicate that physical exercise acutely increases proANP levels in overweight and obese patients, and weight reduction by diet therapy chronically decreases ANP clearance by NPR-C. NP deficiency in obese patients may be ameliorated by the combination of diet and exercise. Increased NP levels stimulate lipolysis [48] and increase lipid oxidation [15] and adiponectin secretion [49] in adipose tissue. NPs regulate thermogenesis in skeletal muscle as well as in adipose tissue [16,50]. Exercise is essential for the management of obesity-associated metabolic and cardiovascular disease, both directly (weight loss) and indirectly (lipolysis by NPs). Two studies have investigated the effects of resistance training on NPs; these studies are different from those mentioned above in that they evaluated the chronic effect of exercise on NT-proBNP rather than the acute effect. The results are controversial. 

Bordbar *et al.* investigated the effects of an eight-week resistance training program on the release of NT-proBNP [51]. A total of 22 subjects were allocated to four groups: sedentary individuals who performed eight weeks of aerobic exercise; sedentary individuals who performed eight weeks of resistance training; bodybuilders who did not use anabolic androgenic steroids; and bodybuilders who regularly used anabolic androgenic steroids. Aerobic exercise was treadmill running, and resistance training consisted of 15 min of machine training and 20 min of isometric exercise using dumbbells. NT-proBNP levels significantly increased immediately after aerobic exercise, but decreased after eight weeks of aerobic exercise. On the other hand, NT-proBNP levels significantly increased after eight weeks of resistance training, although they did not change immediately after resistance training. The authors mentioned the possibility of myocardial damage induced by strength training. 

Beltran Valls *et al.* investigated the effects of 12 weeks of low-frequency, moderate-intensity resistance training on muscle strength and motor function in the elderly [52]. Twenty-eight elderly subjects (mean age = 72 ± 1 years) without cardiovascular, metabolic, pulmonary, or orthopedic diseases were randomly assigned to the training group or control group. Subjects in the training group engaged in resistance training for 12 weeks. They performed the resistance training at an intensity of 40%–50% 1-repetition maximum during the first two weeks, and the resistance was increased approximately every two weeks. Five subjects dropped out of the study. The muscle power of the upper and lower extremities increased by approximately 30% in the training group. Muscle strength also increased by 15%–20% after the 12 weeks of resistance training. However, NT-proBNP concentrations did not change after resistance training. Thus, moderate-intensity resistance training may be a safe and effective strategy for improving muscle fitness in the elderly without inducing cardiac stress. However, a previous cross-sectional study showed that muscle mass is independently and inversely associated with BNP levels [27]; therefore, the change in NPs concentrations due to resistance training may have no relation to NP secretion caused by myocardial stress. 

A total of 1431 healthy middle-aged to elderly subjects were investigated. Thigh muscle mass was evaluated by measuring muscle cross-sectional area using computed tomography. Visceral fat area was also measured using computed tomography at the umbilicus level. Plasma BNP levels were found to be negatively associated with thigh muscle mass and visceral fat area. After adjustment for confounding parameters such as age, gender, body weight, blood pressure, and adiponectin levels, BNP levels were still negatively associated with thigh muscle mass [27]. A previous study in rats showed that BNP protects skeletal muscle mass by decreasing oxidative stress and mitochondrial dysfunction after ischemia-reperfusion [53]. BNP is also secreted from satellite cells within the ischemic skeletal muscle, which can improve the regeneration of neighboring endothelial cells after ischemia. Hence, BNP may also have a protective role against muscle damage in humans. Another cross-sectional study analyzed the association between objectively measured daily physical activity and cardiac biomarkers including NT-proBNP and high-sensitive troponin T in elderly subjects [54]. A total of 1253 community-dwelling subjects (mean age = 75.6 ± 6.5 years) were examined, and their daily physical activity was measured using an accelerometer. After adjustment for age, sex, smoking status, and history of cardiovascular disease, an inverse dose-response relationship with daily walking duration was found for troponin T and NT-proBNP levels. Walking is known to have beneficial effects on cardiovascular morbidity and mortality [55,56], and this study also suggests that daily walking is associated with the reduction of cardiac stress. However, 24.7% of the subjects had a history of cardiovascular disease, and cardiac function tests were not performed; thus, the results should be interpreted carefully. Hamasaki *et al.* showed that objectively measured daily physical activity was positively and independently associated with plasma BNP levels in subjects with glucose intolerance and type 2 diabetic patients [25]. Although this was a small-scale cross-sectional study, light-intensity daily physical activity may also increase BNP levels in patients with metabolic diseases. Table 3 shows a list of published articles investigating the effects of exercise on NPs in the elderly and in patients with metabolic diseases without heart failure.

## 5. The Influence of Exercise Duration and Intensity on NPs Secretion

Differential effects of exercise duration and intensity on exercise-induced NPs secretion should be discussed. Several studies investigating the influence of exercise duration and intensity on the release of cardiac biomarkers have shown that exercise intensity was responsible for the release of cardiac troponin; however, exercise-induced increase of NT-proBNP was associated with exercise duration, but not exercise intensity [57,58,59]. Recently, Benda *et al.* [60] showed that exercise-induced changes in cardiac troponin and BNP is similar between endurance training and a single bout of high-intensity interval training in heart failure patients and healthy controls. Even though exercise is performed at high-intensity, if the duration was short, excessive cardiac stress could be preventive. Although the optimal intensity, frequency, and duration of exercise for health in individuals with and without heart diseases were not fully elucidated, engaging in exercise at above high-intensity and for above a certain duration, which depends on individuals’ physical fitness, may not be beneficial for health.

## 6. Conclusions

In athletes and young healthy subjects, NP concentrations at rest are not elevated, but strenuous endurance exercise significantly increases NP secretion. Even if NP levels exceed the cutoff values, the increase in NPs does not represent myocardial damage, as detected in patients with heart diseases such as myocardial ischemia. Table 4 summarizes the distinct effects of acute exercise, endurance training, and physical deconditioning/disability including older age on NP concentrations. However, further standardized investigations will be required because there are considerable heterogeneities of exercise interventions (intensity, frequency, and duration) between clinical studies.

The exercise-induced increase in NPs may be caused by transient myocardial wall stress, cardiomyocyte metabolic changes, or neuroendocrinological response, which may have cytoprotective and growth-regulating effects on the heart. On the other hand, in the elderly, obese subjects, and patients with metabolic diseases, the physiological mechanism of NP secretion due to exercise seems to be different from that in young, healthy subjects, although NP concentrations also increase during exercise in young, healthy subjects. NP secretion may be more susceptible to cardiac stress in elderly subjects than in young, healthy individuals. In short, exercise-induced increases in NPs may be dependent on the balance between the intensity of exercise and the physical fitness level of the individual. The elderly and patients with metabolic diseases generally have low physical strength; thus, even light-to moderate-intensity exercise could elevate NP levels. However, a number of studies have demonstrated that NP levels are reduced in patients with metabolic diseases including obesity [61,62,63,64], although how exercise contributes to the change in NP levels remains unknown. To elucidate the underlying mechanism of exercise-induced NP secretion in patients with metabolic diseases, further studies are warranted. Recently, the roles of NPs in thermogenesis in fat tissue and oxidative capacity in skeletal muscle have been investigated [14,15,16]. Specifically, BNP may have a protective role for skeletal muscle along with cardioprotective effects in humans. The role of NPs in skeletal muscle beyond a cardiac biomarker will be an issue of study. The acute effects of exercise on NPs have also been investigated in many clinical studies; however, the long-term effects of exercise on NPs are still unknown. Well-designed, prospective studies with large numbers of subjects, preferably RCTs, are needed to elucidate the long-term effects of exercise on NPs in both healthy subjects and patients with metabolic diseases such as type 2 diabetes, hypertension, dyslipidemia, and cardiovascular disease. 

## Figures and Tables

**Table 1:** Effects of exercise on neuroendocrine system.

Hormone Concentrations	Dynamics
Adrenocorticotrophic hormone	↑
Cortisol	↑
Antidiuretic hormone	↑
Growth hormone	↑
Gonadotropins	↓
Testosterone	↓
Estradiol	↓
Natriuretic peptides	↑
Sympathetic nerve system	↑

**Table 2:** Clinical studies investigating the effects of exercise on NP levels in athletes and healthy subjects.

Authors, Year	Subjects	Exercise	Results
Ohba *et al.*, 2001 [29]	10 amateur male athletes, 46.2 ± 10.7 years	100 km ultramarathon	ANP↑, BNP↑ in older athletes ANP↓, BNP↑ in younger athletes
Siegel *et al.*, 2008 [31]	82 healthy runners, 47 ± 8 years	Marathon	BNP→ 4 h after running BNP↑ 24 h after running Creatinine kinase-MB↑, cardiac troponin I↑ both 4 and 24 h after running
Neumayr *et al.*, 2005 [22]	29 healthy recreational cyclists, 34 ± 8 years	Cycle race	NT-proBNP↑ after the race NT-proBNP decreased to baseline values after a week
Scharhag *et al.*, 2005 [32]	105 healthy endurance athletes, 40 ± 8 years	Marathon, 100-km ultramarathon and mountain bike marathon	NT-proBNP↑ Increased NT-proBNP was not associated with increases in cardiac troponin I and T
Scherr *et al.*, 2011 [37]	102 healthy men, 42± 9.5 years	Marathon	NT-pro BNP↑ 0, 24 and 72 h after the race Other cardiac biomarkers returned to normal levels within 72 h
Scharhag *et al.*, 2006 [38]	20 male athletes, 36 ± 7 years	Running or mountain biking 1-h and 3-h exercise with an intensity of 100% and 75% of the individual anaerobic threshold, respectively	NT-proBNP↑, cardiac troponin I↑, cardiac troponin T→ No evidence for myocardial damage was detected after exercise
Faviou, *et al.*, 2008 [39]	43 male soccer and basketball players, 24 ± 4 years, age-matched 21 patients, and 35 healthy controls	Regular training for 11–18 h/week (athletes) Occasional exercise 3–6 h/week (controls)	NT-proBNP↑, cardiac troponin T→

NPs = natriuretic peptides; ANP = atrial natriuretic peptide; BNP = B-type natriuretic peptide; NT-proBNP = N-terminal pro B-type natriuretic.

**Table 3:** Clinical studies investigating the effects of exercise on NP levels in the elderly or in patients with metabolic diseases.

Authors, Year	Study Design	Subjects	Exercise	Results
Engelmann *et al.*, 2005 [45]	Before-after study with control group	38 patients with atrial fibrillation and age-matched 43 older (67.9 ± 4.5 years) healthy controls	Cycle ergometer	ANP↑, BNP↑ during exercise ANP→, BNP→ 30 min after exercise in older healthy controls BNP↓ 30 min after exercise in patients with atrial fibrillation
Tanaka *et al.*, 1995 [46]	Before-after study with control group	19 patients with hypertension (44 ± 2.6 years) and 14 normotensive subjects (40 ± 2.2 years)	Cycle ergometer	ANP↑, BNP↑ during exercise Changes in ANP and BNP levels were associated with only heart rate in hypertensive patients
Haufe *et al.*, 2015 [47]	Randomized, non-controlled trial	125 middle-aged overweight and obese subjects	Cycle ergometer	proANP↑ during exercise proANP→, NPR-C expression↑ after 6-months dietary intervention
Beltran Valls *et al.*, 2014 [52]	Randomized controlled trial	28 elderly subjects, 72 ± 1 years	12-week low-frequency, moderate-intensity resistance training	NT-proBNP→ Muscle strength and power↑

NPs = natriuretic peptides; ANP = atrial natriuretic peptide; BNP = B-type natriuretic peptide; NT-proBNP = N-terminal pro B-type natriuretic; NPR-C = natriuretic peptide receptor C.

**Table 4:** Effects of acute exercise, endurance training, and deconditioning/disability on NP concentrations.

Types of Exercise	at Rest	During or Immediately after Exercise	After (≥72 h) Exercise
**Healthy younger individuals**			
Acute exercise	→	↑	↓ (return to normal levels)
Endurance training	→	→ or ↑	→ or ↓ (return to normal levels)
**Older individuals or Individuals with disabilities or disturbances**			
Older individuals	↗	→ or ↑	↘
Obesity	↘	↑	↓ NPR-C↑
Heart diseases (e.g., heart failure, myocardial infarction and atrial fibrillation)	↑	↑↑	↓ (Appropriate exercise programs may reduce NPs levels less than those at baseline)

NPs = natriuretic peptides; NPR-C = natriuretic peptide receptor C.

## Abbreviations

The following abbreviations are used in this manuscript:

NP	natriuretic peptide
ANP	atrial natriuretic peptide
BNP	B-type natriuretic peptide
NT-proBNP	N-terminal proBNP
RCT	randomized controlled trial
CI	confidence intervals
NPR-C	natriuretic peptide receptor C
